# cTULIP: application of a human-based RNA-seq primary tumor classification tool for cross-species primary tumor classification in canine

**DOI:** 10.3389/fonc.2023.1216892

**Published:** 2023-07-20

**Authors:** Jiaxin Long, Satishkumar Ranganathan Ganakammal, Sara E. Jones, Harish Kothandaraman, Deepika Dhawan, Joe Ogas, Deborah W. Knapp, Matthew Beyers, Nadia A. Lanman

**Affiliations:** ^1^ Department of Biochemistry, Purdue University, West Lafayette, IN, United States; ^2^ Purdue University Institute for Cancer Research, West Lafayette, IN, United States; ^3^ Cancer Science Data Initiatives, Leidos Biomedical Research, Inc., Frederick National Laboratory for Cancer Research, Frederick, MD, United States; ^4^ Department of Veterinary Clinical Sciences, Purdue University, West Lafayette, IN, United States; ^5^ Department of Comparative Pathobiology, Purdue University, West Lafayette, IN, United States

**Keywords:** comparative oncology, deep learning, machine learning, bladder cancer, tumor classification, glioma

## Abstract

**Introduction:**

The domestic dog, *Canis familiaris*, is quickly gaining traction as an advantageous model for use in the study of cancer, one of the leading causes of death worldwide. Naturally occurring canine cancers share clinical, histological, and molecular characteristics with the corresponding human diseases.

**Methods:**

In this study, we take a deep-learning approach to test how similar the gene expression profile of canine glioma and bladder cancer (BLCA) tumors are to the corresponding human tumors. We likewise develop a tool for identifying misclassified or outlier samples in large canine oncological datasets, analogous to that which was developed for human datasets.

**Results:**

We test a number of machine learning algorithms and found that a convolutional neural network outperformed logistic regression and random forest approaches. We use a recently developed RNA-seq-based convolutional neural network, TULIP, to test the robustness of a human-data-trained primary tumor classification tool on cross-species primary tumor prediction. Our study ultimately highlights the molecular similarities between canine and human BLCA and glioma tumors, showing that protein-coding one-to-one homologs shared between humans and canines, are sufficient to distinguish between BLCA and gliomas.

**Discussion:**

The results of this study indicate that using protein-coding one-to-one homologs as the features in the input layer of TULIP performs good primary tumor prediction in both humans and canines. Furthermore, our analysis shows that our selected features also contain the majority of features with known clinical relevance in BLCA and gliomas. Our success in using a human-data-trained model for cross-species primary tumor prediction also sheds light on the conservation of oncological pathways in humans and canines, further underscoring the importance of the canine model system in the study of human disease.

## 1 Introduction

The domestic dog, *Canis familiaris*, is rapidly gaining traction as a useful model with which to study human disease and has been proposed to be a particularly advantageous model in the study of cancer (1–4). Cancer is one of the leading causes of death worldwide and according to the American Cancer Society (https://www.cancer.org/) (5), 1.9 million new cancer cases and over 600,000 cancer-caused deaths were expected to occur in 2022 in the United States (5). Similarly, cancer is one of the leading causes of death in canines, and annually over 4.2 million dogs per year are diagnosed with cancer in the United States (1). The study of naturally occurring tumors in pet dogs through comparative oncology studies has the potential to provide a valuable perspective on tumor biology and a powerful means by which to develop novel therapeutics in both humans and canines.

Comparative oncology is a research field that investigates and compares tumor development and progression across species and (1–4) previous studies in comparative oncology have revealed similarities between naturally-occurring canine and human cancers (1–4). Dogs develop spontaneous tumors which pathologically, biologically, and histologically mirror the formation of tumors in humans. Additionally, canines and humans have numerous shared treatment regimens in various types of cancer (2–4, 6–8). Furthermore, pet dogs share a common living environment with their caregivers, which provides a valuable perspective on how environmental exposures contribute to the development of cancer (1–4). Hence, studies in comparative oncology shed light on basic cancer-related biological pathways. Such studies are also proving to be advantageous for the testing of novel therapeutic targets at the early stages of clinical trials.

**Figure 1:**
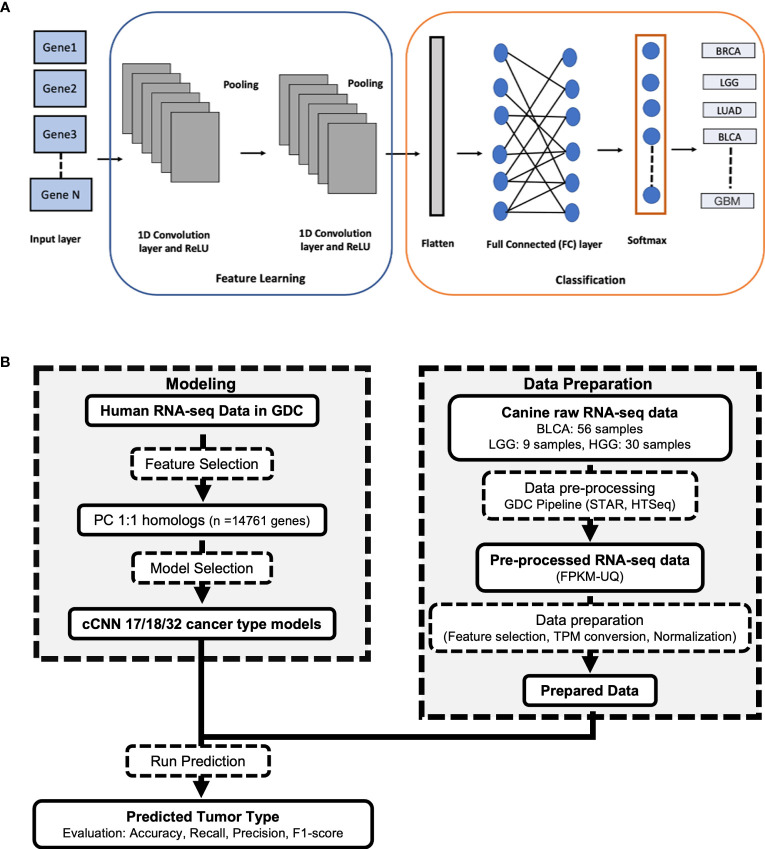
**(A)** Architecture of the TULIP 1D convolutional neural network (CNN) model for classifying RNA-seq samples into different primary tumor types. **(B)** Workflow used in processing the canine RNA-seq data and applying TULIP models to predict primary tumor type.

The rapid generation of omics datasets from studies in oncology coupled with increasingly sophisticated machine learning and deep learning methodologies are ushering in a new era of precision-oncology research. One recently developed tool; the TUmor CLassIfication Predictor (TULIP) is a classification tool that has been developed for the prediction of primary tumor types based on human RNA-seq data (**Figure 1A**) (4). TULIP was trained using normalized RNA-seq data of various human primary tumor types downloaded from the Genomic Data Commons (GDC) (4). All four TULIP models achieve over 95% classification accuracy. Thus, TULIP can be a useful quality control (QC) tool for the identification of misclassified or potential outlier samples in human RNA-seq datasets.

Such a tool would also be useful in identifying any sample-based issues in canine oncological datasets that is analogous to the human sample implementation. Unfortunately, the sample size of canine studies is small in comparison to the large human datasets held in the GDC. As a result, the training of a deep learning model on canine data for the purpose of classifying canine primary tumor types is not practical. Intriguingly, further evaluation of TULIP-derived models on non-TCGA kidney cancer RNA-seq data suggests that the models that were derived from TULIP generalize well to accurately predict the primary tumor types of other non-TCGA data (4).

Several publications have shown that canine and human cancers exhibit clinical, molecular, and histological similarities (2, 9, 10). Here, we take this a step further to see whether the similarities of the transcriptomic profiles are such that a deep learning model trained on human data can accurately classify canine tumors. In this study, we focused on canine bladder cancer and glioma for the initial evaluation of this cross-species tumor type classifier. Both canine bladder cancer and glioma exhibit similar molecular traits to those observed in humans. For example, genes that were identified as dysregulated in human bladder cancer (BLCA) were also identified as such in canine BLCA data (11). Previous genome-wide studies on canine glioma samples suggest that frequently mutated genes that are associated with human glioma are also mutational hotspots in canine glioma samples (10).

Invasive urothelial carcinoma (InvUC) is a highly invasive type of bladder cancer in which tumors grow into the muscle of the bladder (12, 13). Canines have been previously identified and validated as a model for InvUC, and treatment protocols for canine are similar to those used in human patients. Notably, there is a large need for improved therapeutics for the treatment of InvUC in both human and canine (6, 12). InvUC has been identified in 20%-30% of human bladder cancer cases, is the most common bladder cancer type in canines and includes luminal and basal subtypes in both humans and canines (6).

Gliomas are a common type of brain tumor originating in the glial cells that surround and support neurons (14). Human gliomas are classified into 4 grades depending on tumor aggressiveness in the clinic (15). Grades I and II are termed low-grade gliomas (LGG) and grades III and IV are termed high-grade gliomas (HGG) (15). Canine glioma is generally classified into HGG and LGG without the assignment of a numeric grade (16). The study here contains RNA-seq data from two different types of gliomas: oligodendrogliomas and astrocytomas. Oligodendrogliomas originate from oligodendrocytes, and astrocytoma from astrocytes. Clinically, glioblastoma (GBM) is a subset of HGG, specifically a grade IV astrocytoma (17, 18). Several studies have shown that clinical and molecular similarities are exhibited in canine and human gliomas, and particularly between canine LGG and human pediatric glioma (10, 19).

To test how similar the gene expression profile of canine glioma and bladder cancer tumors are to the corresponding human tumors and to identify misclassified or outlier samples in large canine oncological datasets, we sought to determine whether a neural network trained to identify tumor types from human RNA-seq data (20) can accurately predict tumor types in canines. TULIP (20), a 1-dimensional (1D) convolutional neural network (CNN), was used in this cross-species primary tumor classification (**Figure 1A**).

## 2 Materials and methods

### 2.1 RNA-seq data analyses

An overview of the methods used in this study can be found in **Figure 1B**. Fastq files containing raw reads of 56 canine bladder tumor samples (BLCA) and 4 canine normal bladder samples were downloaded from National Cancer Institute’s Integrated Canine Data Commons with the accession ID: 000005 (https://caninecommons.cancer.gov/#/study/UBC02) (6). Fastq files that contain raw reads from 39 canine glioma tumor samples including 30 high-grade glioma (HGG) and 9 low-grade glioma (LGG) samples and 3 normal canine brain samples were downloaded from the NCBI SRA database with the BioProject accession ID: PRJNA579792 (10). Fastq files with raw sequence reads from 5 normal canine bladder samples and 5 normal canine frontal cortex samples were obtained from Barkbase (http://www.barkbase.org) (21), and data were included in the differential gene expression analysis. Raw reads of data from Barkbase were downloaded from the NCBI SRA database with the BioProject accession ID: PRJNA396033 (21). All raw counts were converted to TPM (transcripts per million) to compare the transcript levels of genes across different samples. TPM values of 412 human bladder cancer samples and 711 Glioma samples were downloaded from TCGAbiolinks in BiocManager (Version: 2.12.6) (6, 22, 23).

All RNA-seq data analyses follow the mRNA analysis pipeline established by the National Cancer Institute’s (NCI’s) Genomic Data Commons (GDC) (24). Reads were aligned to the CanFam3.1 reference genome assembly by using STAR v2.7.9a (25). Aligned reads were converted to reads counts using HTseq-count v2.0.1 (26). FPKM-UQ (Fragments per kilobase of transcript per million mapped reads upper quartile) that were calculated using htseq_tools were converted to TPM used to test the performance of the TULIP model on canine model (20, 24).

### 2.2 Detection of the one-to-one homologous genes in human and canine

Human (Grch38.p13) to Canine (CanFam3.1) orthologs were downloaded from the biomaRt query page for Ensembl Genes 104 (27, 28). The fields selected for download were the Gene Stable Id and associated version, Gene Names for both human and dog, Percentage identity of human genes to dog and vice-versa, and Gene-Order Conservation Score with homology type set to dog. Protein coding genes with one-to-one orthologous mapping between human and dog annotations were further extracted and retained for preparing the input files.

### 2.3 Training the cTULIP model

The canine-adapted version of TULIP (cTULIP) is a deep learning Python-based classification tool that utilizes a 1-dimensional (1D) convolutional neural network (CNN) framework (20). It takes human RNA-seq data as the input layer and the output is the predicted primary tumor types with their probability scores. To adapt TULIP for canine tumor type prediction (**Figure 1B**), we obtained RNA-seq data expressed as FPKM-UQ. FPKM-UQ is the upper quartile of the number of fragments per kilobase per million mapped reads. We obtained the FPKM-UQ values for the 9,025 and 9,199 samples corresponding to 17 (sample size > 300 samples) and 18 primary tumor types respectively from the TCGA project in GDC (February 2022) (**Supplementary Table 1**). The 18 primary tumor types include all 17 tumor types with the addition of GBM. We converted the FPKM-UQ values to TPM (transcripts per million) and normalized the TPM values by using a log10 transformation. The data was split randomly into training (80%), validation (10%), and test (10%) datasets using the scikit-learn package (version 1.0.2). The primary tumor types were encoded using the OneHotEncoder() function. We filtered the human protein coding genes to 14,761 genes common between human and canine (one-to-one orthologous mapping). We created two CNN models with Keras (version 2.4.3) that have the number of genes (14,761) as features in the input layer and the number of primary tumor types (17 or 18) in the output layer. The source code is publicly available at https://github.com/CBIIT/CTULIP.

### 2.4 Random forest and logistic regression models

We built random forest (RF) and logistic regression (LR) models using the scikit-learn package for comparing with the cTULIP (*1D-CNN*) models. All parameters were kept at default values. We evaluated the performance of the cTULIP (*1D-CNN*) models along with the random forest (RF) and logistic regression (LR) models using the test dataset by computing the weighted average of precision, recall and F1-score for imbalanced data. The formulas for calculating precision, recall, and F1-score are below.


$$Precision=\frac{TP}{TP+FP}$$

$$Recall=\frac{TP}{TP+FN}$$

$$F1\;score=\frac{2\left(Recall\times Precision\right)}{\left(Recall+Precision\right)}$$

where TP is the number of true positives, TN is the number of true negatives, FP is the number of false negatives, and FN is the number of false negatives.

### 2.5 t-SNE analysis and PCA analysis

A t-distributed stochastic neighbor embedding (t-SNE) was performed by using the Rtsne package v0.15 with perplexity is 4 and 5000 iterations (https://github.com/jkrijthe/Rtsne) (29). The t-SNE plot was visualized by using ggplot2 v3.3.6 (https://ggplot2.tidyverse.org). A principal component analysis (PCA) was performed and visualized with the DEseq2 v1.24.0 package in Bioconductor (30). Both t-SNE plots and PCA analyses were performed by using the top 500 highly variable genes amongst the one-to-one protein coding homologs between the three selected primary tumor types.

### 2.6 Differential gene expression analyses

Differential gene expression analysis was carried out by comparing canine bladder cancer and glioma tumor samples to their corresponding normal samples using the raw count matrices. Differentially expressed genes were identified by using a quasi-likelihood negative binomial generalized linear model from edgeR package v3.26.8 in Bioconductor with a Benjamini-Hochberg false discovery rate< 0.05 (31–33). Volcano plots were generated by using ggplot2 v3.3.6 (https://ggplot2.tidyverse.org) (34).

### 2.7 Identification of clinically relevant genes

Glioma and bladder cancer-associated genes were downloaded from the Online Mendelian Inheritance in Man (OMIM) database (35, 36). Genes associated with either glioblastoma (GBM) or glioma were included in the glioma-associated OMIM genes. Genes linked to either bladder cancer or bladder carcinoma were included in the bladder cancer-associated OMIM genes. In total, 178 BLCA-associated (OMIM) genes (35, 36) were included and 302 glioma-associated genes were included in the intersection analysis. A set of genes that have been previously identified as playing a critical role in both human and canine bladder cancer were included in the clinically relevant genes (6). Intersection analyses between OMIM genes and DEGs were visualized by Venn diagrams and upset plots. Venn diagrams were generated with the VennDiagram R package v1.7.3. Upset plots were generated by using the intersection mode in ComplexHeatmap v2.11.1 (37).

### 2.8 Single-sample gene set enrichment analysis

The enrichment of hallmark signatures (H) and oncogenic signature genes (C6) from the Molecular Signatures Database (MSigDB) on individual samples for all canine bladder cancer data, canine glioma tumor data, human bladder cancer data, and human glioma data was assessed by using a single-sample gene set enrichment analysis (ssGSEA) (38, 39). For the sake of clear visualization and to achieve balanced sample numbers across cancer types, we randomly selected 9 samples from each canine cancer and 10 samples from each human primary tumor type. The ssGSEA derived scores were plotted in heat maps and grouped based on the primary tumor types and species. Only protein-coding genes with one-to-one homologous mapping between human and canine data were included for ssGSEA. TPM values of human bladder cancer data and glioma data were downloaded from TCGAbiolinks in BiocManager (Version: 2.12.6) (6, 22, 23). TPM values of canine data were calculated from the count matrix generated from HTseq-count v2.0.1 (26). The significant gene sets were selected with a false discovery rate threshold of< 0.05.

## 3 Results

### 3.1 Protein-coding one-to-one homologs are sufficient for classifying primary tumor types in canine

**Figure 2:**
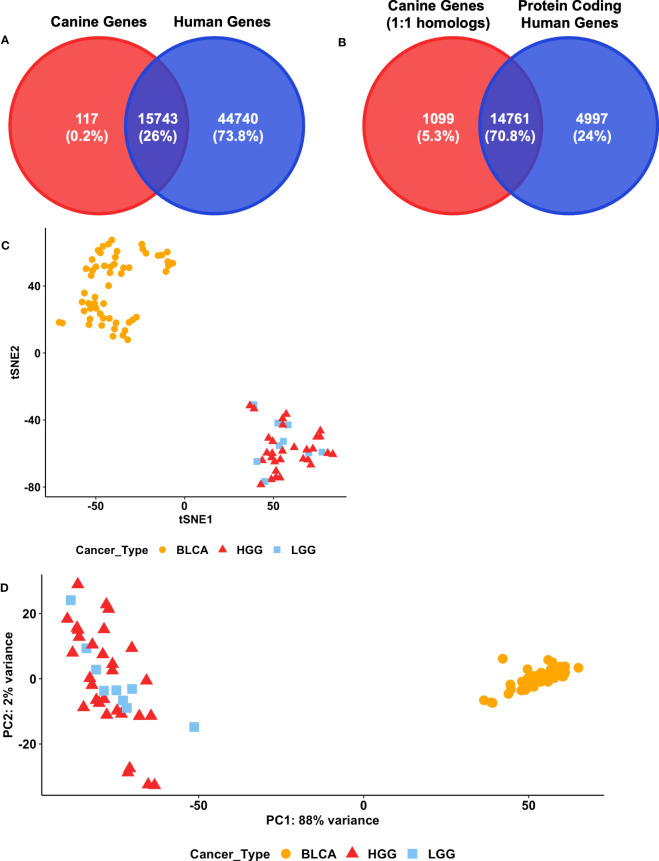
Feature selection for modeling. **(A)** Venn diagrams of the intersection between the 15860 annotated canine genes that have one-to-one human homologs and the 60483 human genes annotated in the GDC. **(B)** Venn diagrams of the intersection between 15860 annotated canine genes that have one-to-one human homologs and 19758 annotated protein coding human genes in GDC. **(C)** t-SNE analysis of three cancer types in canine datasets. The 500 most variable of the 14761 canine genes that have one-to-one protein coding human homologs in the GDC were used. BLCA = Bladder cancer, LGG = Lower-grade glioma, HGG = High-grade glioma. **(D)** Principal component analysis of canine bladder cancer and glioma datasets. The 14761 canine genes that have one-to-one protein coding human homologs in the GDC were included. The top 500 genes exhibiting the highest row variance were used in this analysis. BLCA, Bladder cancer; LGG, Lower-grade glioma; HGG, High-grade glioma.

The selection of features is a critical first step to enable the use of a cross-species classifier on canine data. Initially, one-to-one homologs between canine and human genes were selected as the features for the input layer of the models (**Figure 1B**). Overall, 15,860 canine genes have one-to-one homologs in the human genome (**Figure 2A**). In addition, the initial classification of TULIP on human data suggests that only using protein-coding genes as the input layer is also sufficient to perform primary tumor type prediction in that the model achieves 97.6% accuracy (20). A total of 14,761 out of 15,860 genes that have one-to-one human homologs in canine were also identified as protein-coding genes in the human genome (**Figure 2B**).

To visually inspect whether protein-coding one-to-one homologs are sufficient to distinguish between primary tumor types in canine, we employed t-SNE and a PCA on canine glioma and bladder cancer RNA-seq data (**Figures 2C, D**). Both analyses suggest that protein-coding one-to-one homologs are sufficient to distinguish between canine glioma and bladder cancer tumor samples (**Figures 2C, D**; **Supplementary Figures 1A, B**). However, these genes failed to distinguish between canine high-grade glioma (HGG) and low-grade glioma (LGG) samples.

Next, we investigated whether a model trained on human data using protein-coding one-to-one homologs would provide more robust predictive power than a model using all one-to-one homologs. We first compared the performance of the models on human data with the two selected feature sets and used the 17 primary tumor types as the output layer (**Supplementary Table 1**). The model using protein-coding genes (*cCNN-17-PC*) achieves an accuracy of 96.2% whereas the model with all one-to-one homologs (*cCNN-17*) achieves an accuracy of 95.7%. Ultimately, the model trained with protein-coding one-to-one homologs achieves a higher accuracy, precision, recall, and F1 score than the model trained on all one-to-one homologs (**Table 1**). This observation is consistent with that previously observed in the human study (20). Therefore, we continued our analysis by using protein-coding one-to-one homologs as the selected features for the input layer.

**Table 1:** Summary of the performance evaluation of the various models on human data.

Features	Precision	Recall	F1-score	Accuracy
17 PRIMARY TUMOR TYPES (Total samples: 9025)				
cCNN-17-PC	96.2%	96.1%	0.961	96.2%
cCNN-17	96.0%	95.7%	0.957	95.7%
18 PRIMARY TUMOR TYPES (Total samples: 9199)				
cCNN-18-PC	96.6%	96.4%	0.965	96.5%
32 PRIMARY TUMOR TYPES (Total samples: 10940)				
cCNN-32-PC	91.6%	92.2%	0.915	92.3%

Accuracy, recall, precision and F1 score were used to quantify the performance of each model. The total number of human RNA-seq samples and selected features that were included in each dataset used to train various models are indicated. PC 1:1 homologs included the 14761 protein coding human genes with one-to-one homologs in the canine genome. The 1:1 homologs included the 15743 human genes with one-to-one homologs in the canine genome. Primary tumor types included in the models are provided in **Supplemental Tables 1-3**.

### 3.2 Model selection for cross-species cancer type prediction

In addition to the features used in the input layer, the number of primary tumor types in the output layer also impacts the model performance (20). The initial development of TULIP allows users to choose either a 17 or a 32 primary tumor type model (**Supplementary Tables 1, 2**). Glioblastoma (GBM) was not included in the 17 primary tumor type model due to the relatively small number of samples available through TCGA, but it was included in the 32 primary tumor type model (**Supplementary Table 2**). We used protein-coding one-to-one homologs as features in the input layer and tested the classification performance of the models on human data. For the sake of simplicity, we refer to the 17 primary tumor type model as the *cCNN-17-PC* model and the 32 primary tumor type model as the *cCNN-32-PC* model in the remainder of the manuscript (**Table 1**). Both models achieve an accuracy that is greater than 92% when used with human testing datasets (**Table 1**). The *cCNN-17-PC* model performs well on predicting the primary tumor types in human data sets and outperformed the *cCNN-32-PC* model (*cCNN-17-PC*: accuracy: 96.2%, precision: 96.2%, recall: 96.1%, F1 score: 0.961; *cCNN-32-PC*: accuracy: 92.3%, precision: 91.6%, recall: 92.2%, F1 score: 0.915) (**Table 1**). To examine the ability of TULIP to distinguish between glioma tumor grades in both human and canine, we constructed an 18 primary tumor type model (*cCNN-18-PC* model) that includes the primary tumor types in the 17 primary tumor type model with the addition of glioblastoma (**Supplementary Table 3**). The *cCNN-18-PC* model also performs very well on predicting primary tumor type (*cCNN-18-PC*: accuracy: 96.5%, precision: 96.6%, recall: 96.4%, F1 score: 0.965). Both the *cCNN-17-PC* and the *cCNN-18-PC* models outperformed the *cCNN-32-PC* model with an accuracy of greater than 96% (**Table 1**). In addition, both the *cCNN-17-PC* and the *cCNN-18-PC* models accomplish greater than 95% for precision, recall, and F1 score.

To further benchmark the performance of cTULIP (1D-CNN) models with other standard machine learning algorithms, we constructed random forest (RF) and logistic regression (LR) models with the 17 and 18 primary tumor types. The comparison indicates that the accuracy of the 1D-CNN models surpassed both the RF and LR models in all metrics (**Table 2**).

**Table 2:** Performance of various training algorithms on human data.

Statistics	cCNN-17-PC			cCNN-18-PC		
	CNN	RF	LR	CNN	RF	LR
Precision	96.2%	92.0%	82.0%	96.6%	89.0%	80.0%
Recall	96.1%	90.0%	83.0%	96.4%	90.0%	81.0%
F1-score	0.961	0.890	0.820	0.965	0.890	0.800
Accuracy	96.2%	90.0%	83.0%	96.5%	90.0%	81.0%

Accuracy, recall, precision and F1 score were used to quantify the performance of each model. The 14761 protein coding genes that have one-to-one homologs in the human and canine genomes were used in the model input layer. The 17-cancer-type model included 17 primary tumor types as annotated in the GDC and the 18-cancer-type model included 17 primary tumor types as indicated previously with the addition of glioblastoma (**Supplemental Tables 1–3**). CNN, Convolutional Neural Network; RF, Random Forest; LR, Logistic Regression.

### 3.3 Cross-species primary tumor classification performance

According to the performance on human data, both the *cCNN-17-PC* and the *cCNN-18-PC* models classify human primary tumor types robustly (**Table 1**). Next, we evaluated the predictive power of these two human data-trained models on canine data. We tested whether the *cCNN-17-PC* model can robustly classify canine bladder cancer and canine glioma samples. Overall, this model exhibits good predictive power in that it achieves an accuracy of 75.8%, a recall of 0.758, and an F1 score of 0.867 (**Table 3**). Without distinguishing between the grades of glioma tumors, the *cCNN-18-PC* model performed better than the *cCNN-17-PC* with an accuracy of 80.0%, a recall of 0.800, and an F1 score of 0.889 (**Table 3**). Thus, including GBM in the model results in a somewhat improved performance relative to the *cCNN-17-PC* model. We also tested the ability of the model to distinguish between the grades of glioma tumors in canine using the *cCNN-18-PC* model. However, this model fails to accurately predict the grades of primary tumors (**Table 3**). In particular, the accuracy drops to 48.4%, and precision, recall, and F1 score decrease significantly as well. The inspection of individual canine samples reveals that even though an increased number of canine bladder cancer samples are classified into BLCA correctly by using the *cCNN-18-PC* model, very few HGG samples have been assigned to GBM (**Supplementary Table 2**). Overall, the human data-trained models can classify canine primary tumor types, but further optimization is needed to distinguish between tumor grades.

**Table 3:** Summary of performance evaluation of various models on canine data.

Models	Precision	Recall	F1-score	Accuracy	Notes
Canine test data (Total samples: 95)					
cCNN-17-PC	100.0%	75.8%	0.862	75.8%	
cCNN-18-PC	100.0%	80.0%	0.889	80.0%	Regardless of the grades of glioma samples
cCNN-18-PC	61.3%	48.4%	0.541	48.4%	

Precision, Recall, F1-score and accuracy were calculated on the selected canine RNA-seq data. A total of 95 canine samples were included. Primary tumor types included in the models are provided in **Supplemental Tables 1–3**.

### 3.4 Selected features capture key genes of bladder cancer and glioma

**Figure 3:**
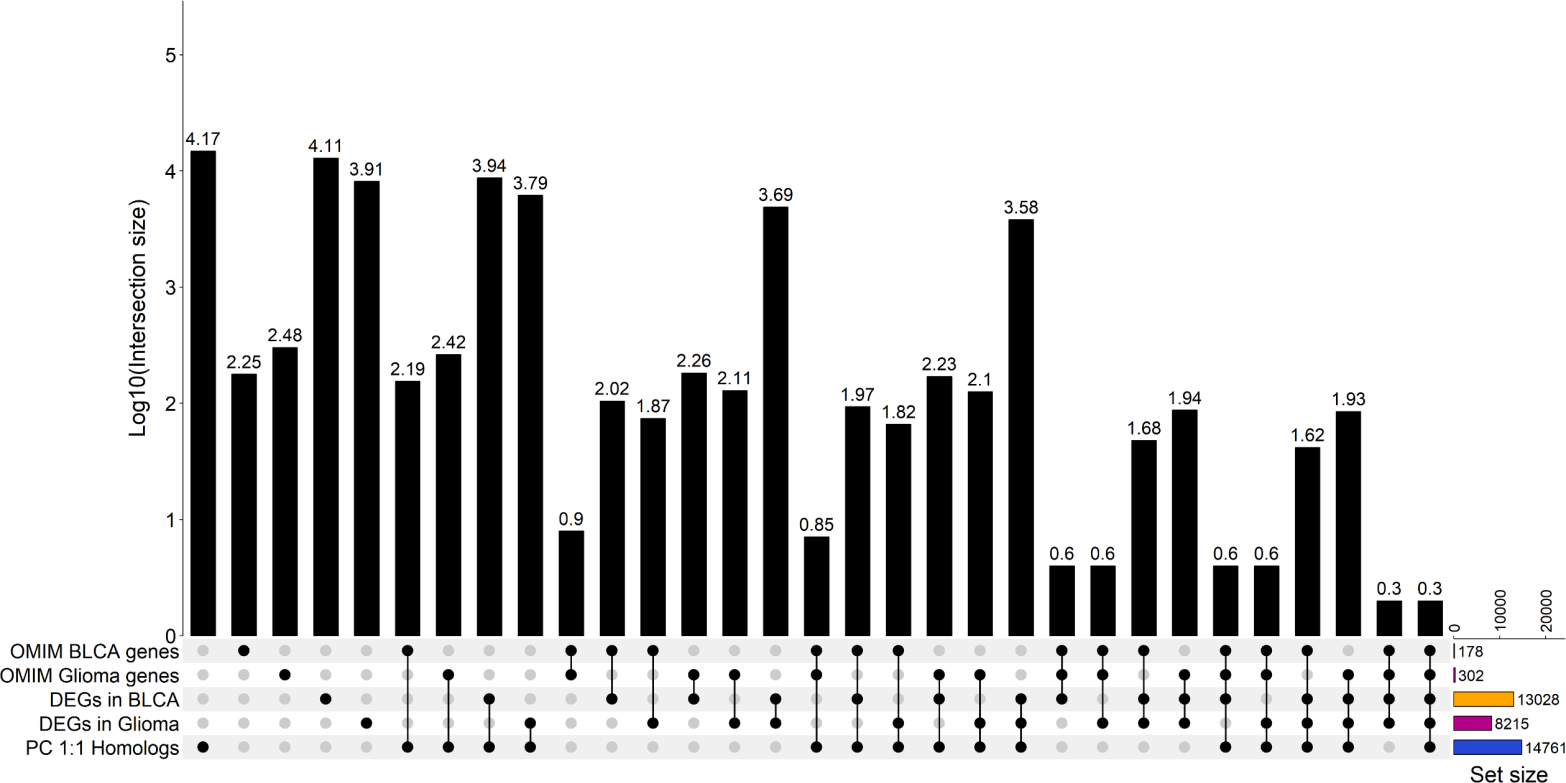
Upset plot showing the summary of the intersection between bladder cancer and glioma OMIM genes with selected features that were included in the model input layers. Intersection analysis between gene sets of significantly differentially expressed genes in each cancer type as indicated, human genes associated with each cancer type (OMIM genes) and annotated protein-coding human genes as indicated previously. The OMIM BLCA gene set is the union of genes associated bladder cancer and bladder carcinoma. The OMIM glioma genes set is the union of genes associated with glioma and glioblastoma [32,33]. Significantly differentially expressed genes of each canine cancer type were identified relative to normal samples with false discovery rate (FDR)< 0.05. DEGs in glioma were identified by using all low-grade glioma and high-grade glioma samples relative to normal samples. The total number of genes in each set is indicated by the set size. A black dot indicates the data sets that were intersected; a grey dot shows that a gene set is excluded from the given intersection analysis. The size of the intersection between gene sets is shown on a log_10_ scale as annotated at the top of each bar.

**Figure 4:**
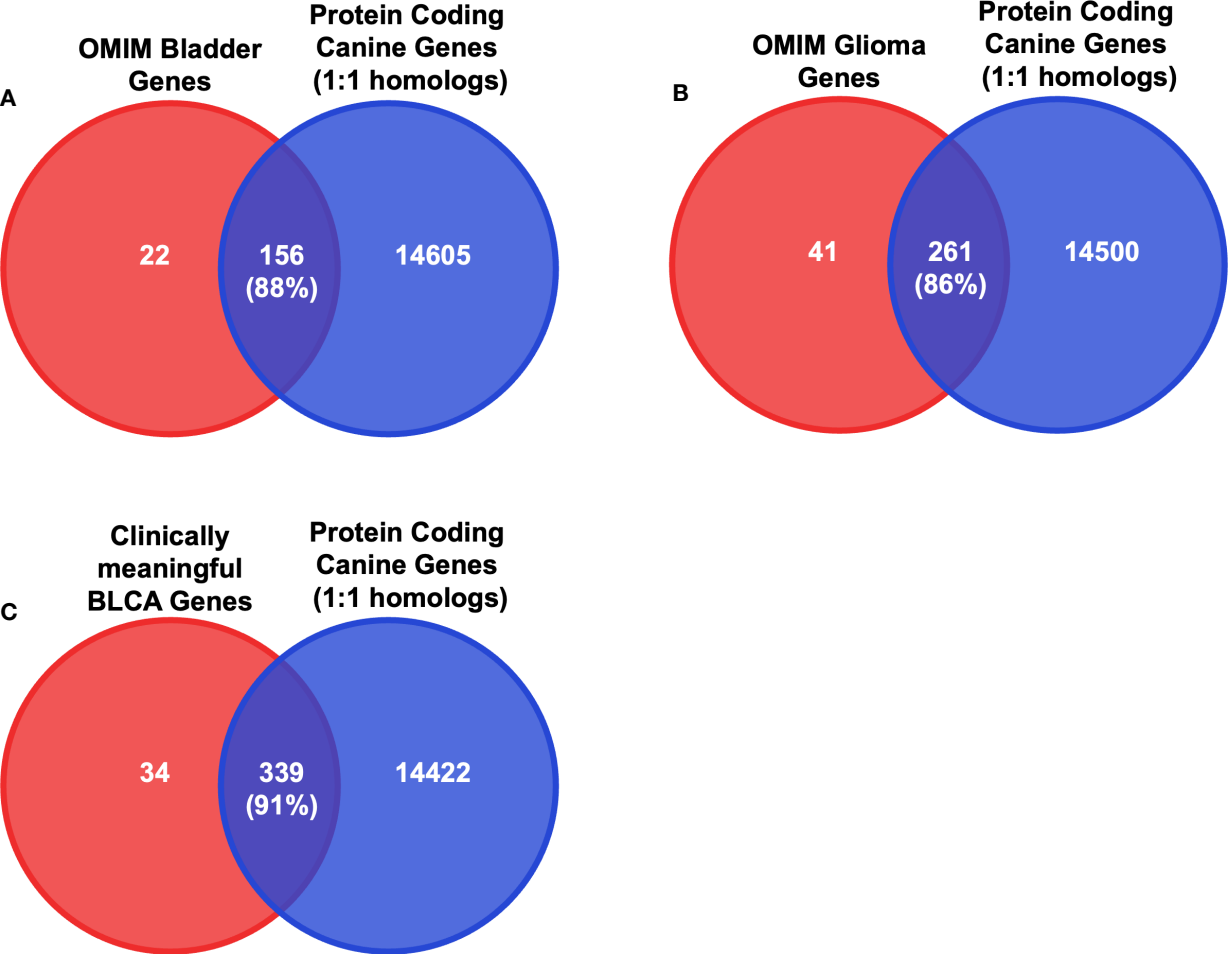
Intersection analysis between critical BLCA and glioma genes and protein-coding one-to-one homologs. **(A)** Diagrams of the intersection between the 14761 protein-coding canine genes that are one-to-one homologs and the bladder cancer-associated OMIM genes. Numbers in parentheses indicate the percentage of the intersection of OMIM bladder genes. **(B)** Diagrams of the intersection between the 14761 protein-coding canine genes that are one-to-one homologs and the glioma-associated OMIM genes. Numbers in parentheses indicate the percentage of the intersection of OMIM glioma genes. **(C)** Diagrams of the intersection between the 14761 protein-coding canine genes that are one-to-one homologs and identified clinically meaningful genes in both human and canine bladder cancers. Numbers in parentheses indicate the percentage of the intersection of clinically meaningful BLCA genes.

Since we selected only the one-to-one protein-coding homologs for use in the input layer, we sought to determine the cancer relevance of these genes in humans. We compared these genes to those with established importance in either human bladder cancer or human glioma (**Figure 3** and **Supplementary Tables 5, 6**) from the Online Mendelian Inheritance in Man (OMIM) database (35, 36). In total, there are 178 BLCA-associated OMIM genes and 302 glioma-associated OMIM genes that have canine homologs. Approximately 88% of BLCA-associated OMIM genes and 86% of glioma-associated OMIM genes were included as features in the input layer (**Figures 3**, **4A, B** and **Supplementary Tables 5, 6**). In addition, we compared the selected features to 402 genes that were previously shown to be clinically relevant in bladder cancer (6, 40, 41). Of the 402 genes, 373 have at least one canine homolog and 339 are protein-coding one-to-one homologs (**Figure 4C** and **Supplementary Table 7**).

**Figure 5:**
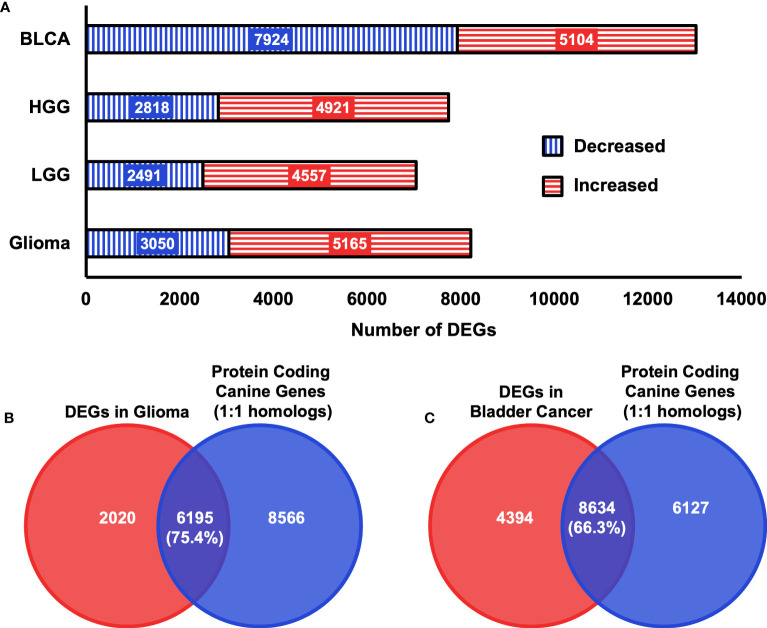
Summary of DEGs in two canine cancer datasets. **(A)** Total numbers of differentially expressed genes that were identified in each canine cancer type as indicated relative to normal samples with a false discovery rate threshold of< 0.05. BLCA = Bladder cancer, LGG = Low-grade glioma, HGG = High-grade glioma, Glioma = High-grade glioma and low-grade glioma. **(B)** Diagrams of the intersection between 14761 protein coding canine genes that are one-to-one homologs and identified DEGs in canine Glioma samples. Numbers in parentheses indicate the percentage of intersection of DEGs identified in canine glioma samples. **(C)** Diagrams of the intersection between 14761 protein-coding canine genes that are one-to-one homologs and identified DEGs in canine bladder cancer samples. Numbers in parentheses indicate the percentage of the intersection of DEGs identified in canine bladder cancer samples.

**Figure 6:**
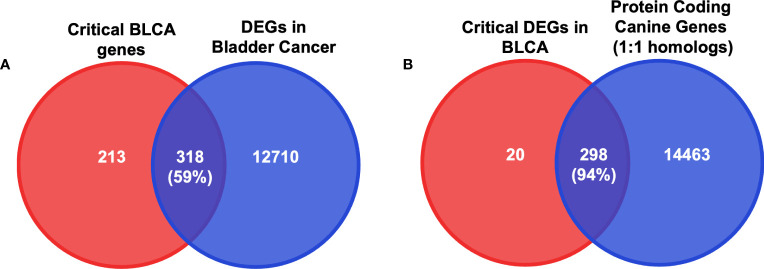
DEGs in BLCA in canine RNA-seq data. **(A)** Diagrams of the intersection between critical bladder-cancer-associated genes and DEGs in canine bladder cancer samples. Numbers in parentheses indicate the percentage of the intersection of critical BLCA genes. **(B)** Diagrams of the intersection between critical DEGs in canine bladder cancer samples and protein-coding one-to-one homologs. Numbers in parentheses indicate the percentage of the intersection of critical BLCA genes that are identified as DEGs in canines.

Next, we examined whether the selected features used in the input layer are biologically relevant in canine tumors. To this end, we performed a differential expression analysis in both canine bladder cancer and canine glioma compared to their corresponding normal samples. In total, 13,028 differentially expressed genes (DEGs) were identified in canine bladder cancer tumors and 8,215 DEGs were identified in canine glioma tumors (**Figure 5A**). Over 66% of DEGs in bladder cancer tumors and over 75% of DEGs in glioma tumors are protein-coding one-to-one homologs (**Figures 3**, **5B, C**). Furthermore, we combined the 402 clinically meaningful genes that were identified from previous whole-genome sequencing and RNA-seq analyses (40, 41) and bladder cancer-associated OMIM genes that contain known BLCA drivers to generate a list of 535 genes with documented importance in bladder cancer. A total of 318 critical BLCA genes are also differentially expressed in canine bladder cancer samples (**Figure 6A**), 289 of which were included as features in the input layer (**Figure 6B**). By and large, the selected features included in the input layer appear to capture most genes that are relevant in canine and human bladder cancer and glioma.

### 3.5 Transcriptomic profiles alone may not be sufficient for the classification of canine glioma grades

The evaluation of these human-trained models suggests that TULIP can perform cross-species primary tumor classification, however, it cannot accurately distinguish between grades of canine glioma tumors (**Table 3**). This observation is also supported by **Figures 2C, D**. To further investigate possible causes of this caveat, we undertook a series of analyses as described below.

**Figure 7:**
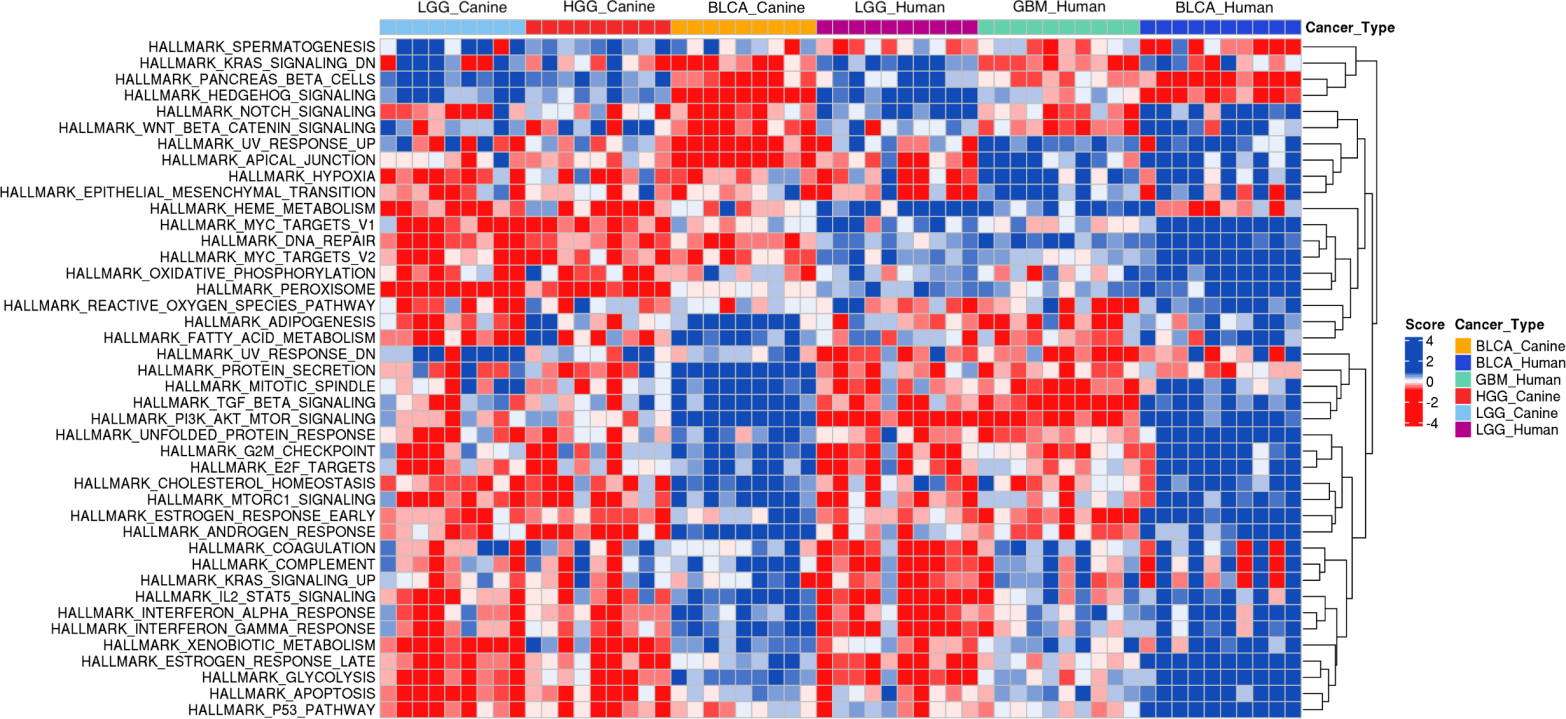
Single-sample Gene Set Enrichment Analysis of canine RNA-seq data and human RNA-seq data. Annotated hallmark gene sets in the MSigDB database were included in the analysis. The heat map of derived ssGSEA scores of 9 randomly selected samples of each canine cancer type and 10 randomly selected randomly samples from each human primary tumor type are shown. Enriched gene sets in either canine glioma or canine bladder cancer were determined by having at least 2/3 of total sample size with an FDR threshold of< 0.05. The union of enriched gene sets in both cancer types results in 42 gene sets as indicated. BLCA, Bladder cancer; LGG, Low-grade glioma; GBM, Glioblastoma; HGG, High-grade glioma.

First, we performed a single-sample gene set enrichment analysis (ssGSEA) to examine whether the one-to-one protein-coding homologs that are used in the input layer of the model recapitulate the human glioma and bladder cancer molecular signatures in the canine data. As the heat maps show, glioma and bladder cancer exhibit distinct gene expression patterns (**Figure 7**; **Supplementary Figures 2, 3**). Intriguingly, the hierarchical clustering reveals that canine LGG and HGG samples share very similar transcriptomic signatures, and that canine glioma exhibits a more similar gene expression pattern to human LGG than to GBM. Moreover, canine and human bladder cancer samples exhibit similar gene expression patterns to one another (**Figure 7**; **Supplementary Figure 3**).

**Figure 8:**
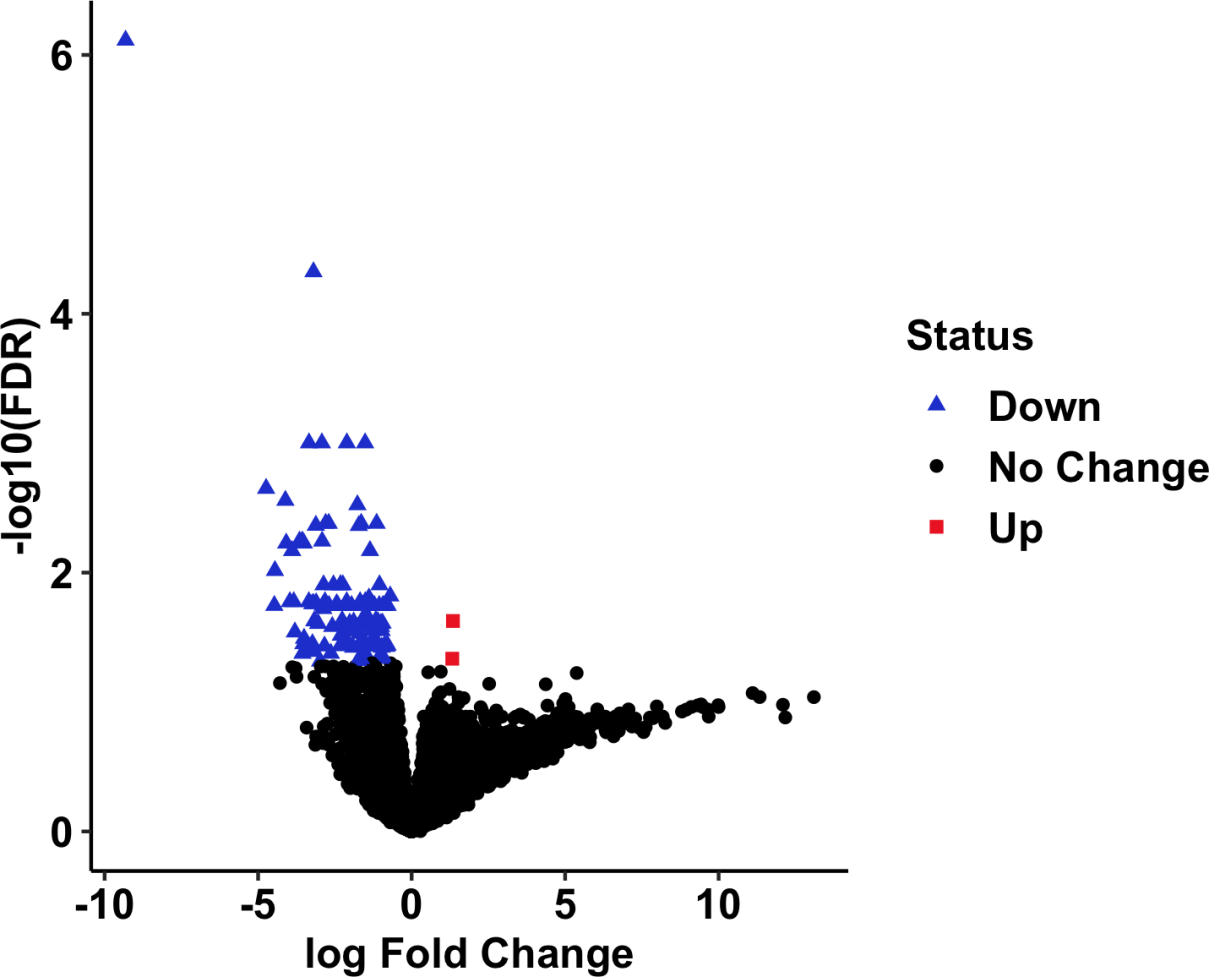
DEGs in HGG vs. LGG in canine RNA-seq data. Significantly differentially expressed genes were identified in HGG relative to LGG with a false discovery rate threshold of< 0.05. Significantly downregulated genes were colored by blue, and significantly upregulated genes were colored by red. LGG, Low-grade glioma; HGG, High-grade glioma.

We also performed a differential expression analysis between HGG and LGG in canine. Only 117 DEGs were identified (**Figure 8**), suggesting that there may be few enough transcriptomic differences between grades of canine glioma to render classification using gene expression profiles alone to be inadequate, which could contribute to the low classification power observed in segregating the LGG and HGG samples.

## 4 Discussion

The canine model has in recent years been gaining traction as a valuable system for studying a number of human diseases, including cancer (1–4). This study highlights the transcriptomic similarities between canine bladder cancer and glioma with the corresponding human diseases, and in doing so underscores the importance of pet dogs as a translational model in oncology. In this study, we find that TULIP (TUmor CLassIfication Predictor), a deep learning classification tool trained on RNA-seq data from human tumors, can classify canine primary tumor types (20). The developed *cCNN-17-PC* model performs good classification of canine tumor types with an accuracy of 75.8%, a recall of 75.8% and an F1 score of 0.862, likewise the *cCNN-18-PC* model achieves an accuracy of 80%, a recall of 80.0% and an F1 score of 0.889.

Additionally, the feature selection performed in this study found that protein-coding genes that have one-to-one homologs in the human and canine genome are sufficient to distinguish primary tumor types in both human and canine. On the human test dataset, the *cCNN-17-PC* model, which uses protein-coding one-to-one homologs as features in the input layer outperforms the *cCNN-17* model that uses all one-to-one homologs. The improved performance of models using protein-coding one-to-one homologs indicates that most biologically relevant genes were included as features in the input layer. The implication of this observation is that the basis of oncogenesis is fundamentally evolutionarily conserved. This finding provides independent evidence to support the use of canines as a relevant model for human cancers. It likewise suggests that the current selection of input genes for the 1D-CNN models also includes the critical genes for distinguishing between primary tumor types.

It should be noted here that strictly speaking our analysis doesn’t rule out the alternative hypothesis that even though the initial oncogenic pathways are conserved, the subsequent progression pathways may have diverged in humans and canines. However, the observed conservation of oncogenes and oncogenic pathways, as well as the ability to classify primary tumor types in canines with a human-data-trained model suggest otherwise. Specifically, a differential expression analysis and comparison to genes with known cancer relevance support the hypothesis that protein-coding one-to-one homologs include most of the known cancer-relevant genes in canine and human. In addition, the ssGSEA provides further evidence that the canine and human diseases share strikingly similar transcriptomic profiles, including those associated with oncogenic signaling and hallmark pathways.

Furthermore, we found that 1D-CNN models that were derived from TULIP also outperformed other well-known machine learning algorithms, both the *cCNN-17-PC* and *cCNN-18-PC* models achieved accuracies of at least 96% while the accuracies of the 17 and 18 primary tumor type random forest and logistic regression models had accuracies of at least 90% and 85% respectively.

This study also highlights, as in the previous study (20) that the smaller c*CNN-17-PC* and c*CNN-18-PC* models outperform the c*CNN-32-PC* model. We strongly suspect that the performance gap between the *CNN-32-PC* model and the smaller models is due to the number of samples available for the various types of cancer in the Genomic Data Commons. In particular, the BLCA and LGG datasets each contain more than 400 samples, however, the GBM dataset only contains 174 samples in the training data. The previous study on the initial development of TULIP has demonstrated that the class imbalance of primary tumor types in the training dataset has a significant impact on the accuracy of the model (20).

TULIP has previously been shown to enable the identification of different types of primary kidney tumors, kidney renal clear cell carcinoma and kidney renal papillary cell carcinoma, that originate in the same organ (20). This observation raises the possibility of using this model to classify samples by glioma grades. Even though the *cCNN-18-PC* model is ultimately not able to perform robust classification between grades of canine glioma tumors, there is potential that this model could classify glioma tumor grade in canines with further optimization. For example, there is a fundamental discrepancy in the pathological classification of grades of glioma in canines and humans that could impact the predictive power of the *cCNN-18-PC* model. Canine gliomas are classified into HGG and LGG without an assignment of a numeric grade (16). In contrast, human LGG samples stands for lower grade glioma, including grades II and III glioma (42), and GBM samples are grade IV astrocytomas, which have a distinct clinical and molecular characterization (15, 18). In addition, canine HGG samples contain both oligodendrogliomas and astrocytomas, and the majority of samples are oligodendrogliomas (10). In particular, the ssGSEA analysis indicates that the biological pathways that are overrepresented in canine LGG and HGG largely mirror the representative biological pathways in human LGG samples while human GBM samples exhibit a distinct enrichment of biological pathways. It is not hard to speculate that the class imbalance of primary tumor types could contribute to the lower predictive power of the *cCNN-18-PC* model in distinguishing between grades of canine gliomas. Therefore, future training for cross-species machine learning with a balanced number of samples sharing more similar transcriptomic signatures might be required to improve model performance.

According to the differential expression analysis, few differentially expressed genes were identified between canine LGG and HGG which may also contribute to the low predictive power of the *cCNN-18-PC* model for glioma grade prediction. However, a caveat is that we suspect that the low number of canine LGG samples (n=9) is negatively impacting our ability to identify DEGs. However, our study only highlights the similarity of the transcriptomic profiles and the cancer relevance of selected features in both canine and humans. Previous studies on canine methylation patterns reveal that different grades of canine glioma exhibit distinct DNA methylation profiles (10). This finding suggests that the inclusion of epigenomic profiles might improve the performance of TULIP in glioma grade classification.

In conclusion, this study underscores the similarities between the gene expression profiles of canine bladder cancer and canine glioma with that of the corresponding human diseases. This study also highlights a general cross-species primary tumor classification pipeline by developing 1D-CNN models for primary tumor type prediction in humans and in canines. This is, to our knowledge, the first example of a cross-species machine-learning primary tumor type predictor. At the same time, this study also sheds light on the need of additional multi-omics analysis in comparative oncology. This study highlights the translational potential of the canine model system, and ultimately, paves the way for the development of more advanced cross-species machine-learning models with multi-omics sequencing analysis that could have practical clinical applications, such as tumor subtype identification as well as analysis of the impact of novel therapies.

## Data availability statement

Publicly available datasets were analyzed in this study. This data can be found here: The datasets analyzed in this study can be found in the NCBI SRA database with the BioProject accession IDs: PRJNA579792 (https://www.ncbi.nlm.nih.gov/bioproject/?term=PRJNA579792) and PRJNA396033 (https://www.ncbi.nlm.nih.gov/bioproject/PRJNA396033) or at the National Cancer Institute’s Integrated Canine Data Commons with the accession ID: 000005 (https://caninecommons.cancer.gov/#/study/UBC02).

## Author contributions

MB, NL, SG, and JL contributed to the conception and design of the study. SG, JL, HK, and SJ performed deep learning, bioinformatics, and statistical analyses. NL, DD, JO, and DK identified datasets and comparative measures as well as provided biological context. JL and NL wrote the first draft of the manuscript. MB, SJ, SG, and HK wrote sections of the manuscript. MB, NL, SG, SJ, HK, and JL interpreted the data. All authors contributed to the article and approved the submitted version.

## References

[1] Schiffman JD Breen M. Comparative oncology: what dogs and other species can teach us about humans with cancer. Philos Trans R Soc Lond B Biol Sci (2015) 370:20140231. doi: 10.1098/rstb.2014.02311 26056372 PMC4581033

[2] Gardner HL Fenger JM London CA. Dogs as a model for cancer. Annu Rev Anim Biosci (2016) 4:199–222. doi: 10.1146/annurev-animal-022114-1109112 26566160 PMC6314649

[3] Shearin AL Ostrander EA. Leading the way: canine models of genomics and disease. Dis Model Mech (2010) 3(1-2):27–34. doi: 10.1242/dmm.0043583 20075379 PMC4068608

[4] Cadieu E Ostrander EA. Canine genetics offers new mechanisms for the study of human cancer. Cancer Epidemiol Biomarkers Prev (2007) 16(11):2181–3. doi: 10.1158/1055-9965.EPI-07-26674 17982116

[5] American Cancer Society. Cancer statistics center (2023). Available at: http://cancerstatisticscenter.cancer.org (Accessed 2023).

[6] Sommer BC Dhawan D Ruple A Ramos-Vara JA Hahn NM Utturkar SM. Basal and luminal molecular subtypes in naturally-occurring canine urothelial carcinoma are associated with tumor immune signatures and dog breed. Bladder Cancer (2021) 7:1493(3). doi: 10.3389/fonc.2019.01493 PMC11181872 38993617

[7] Aresu L Ferraresso S Marconato L Cascione L Napoli S Gaudio E. New molecular and therapeutic insights into canine diffuse large b-cell lymphoma elucidates the role of the dog as a model for human disease. Haematologica (2019) 104(6):E256–E9. doi: 10.3324/haematol.2018.2070277 PMC6545862 30545928

[8] Prouteau A Andre C. Canine melanomas as models for human melanomas: clinical, histological, and genetic comparison. Genes (Basel) (2019) 10(7):501. doi: 10.3390/genes100705018 31262050 PMC6678806

[9] Mitchell D Chintala S Fetcko K Henriquez M Tewari BN Ahmed A. Common molecular alterations in canine oligodendroglioma and human malignant gliomas and potential novel therapeutic targets. Front Oncol (2019) 9:7809. doi: 10.3389/fonc.2019.007809 PMC6702544 31475119

[10] Amin SB Anderson KJ Boudreau CE Martinez-Ledesma E Kocakavuk E Johnson KC. Comparative molecular life history of spontaneous canine and human gliomas. Cancer Cell (2020) 37(2):243–57. doi: 10.1016/j.ccell.2020.01.00410 PMC7132629 32049048

[11] Parker HG Dhawan D Harris AC Ramos-Vara JA Davis BW Knapp DW. RNAseq expression patterns of canine invasive urothelial carcinoma reveal two distinct tumor clusters and shared regions of dysregulation with human bladder tumors. BMC Cancer (2020) 20(1):251. doi: 10.1186/s12885-020-06737-011 32209086 PMC7092566

[12] Czerniak B Dinney C McConkey D. Origins of bladder cancer. Annu Rev Pathol (2016) 11:149–74. doi: 10.1146/annurev-pathol-012513-10470312 26907529

[13] Malkowicz SB van Poppel H Mickisch G Pansadoro V Thuroff J Soloway MS. Muscle-invasive urothelial carcinoma of the bladder. Urology (2007) 69(1):3–16. doi: 10.1016/j.urology.2006.10.04013 17280906

[14] Weller M Wick W Aldape K Brada M Berger M Pfister SM. Glioma. Nat Rev Dis Primers (2015) 1:15017. doi: 10.1038/nrdp.2015.1714 27188790

[15] Louis DN Holland EC Cairncross JG. Glioma classification: a molecular reappraisal. Am J Pathol (2001) 159(3):779–86. doi: 10.1016/S0002-9440(10)61750-615 PMC1850454 11549567

[16] Koehler JW Miller AD Miller CR Porter B Aldape K Beck J. A revised diagnostic classification of canine glioma: towards validation of the canine glioma patient as a naturally occurring preclinical model for human glioma. J Neuropathol Exp Neurol (2018) 77(11):1039–54. doi: 10.1093/jnen/nly08516 PMC6181180 30239918

[17] Taylor OG Brzozowski JS Skelding KA. Glioblastoma multiforme: an overview of emerging therapeutic targets. Front Oncol (2019) 9:96317. doi: 10.3389/fonc.2019.0096317 PMC6775189 31616641

[18] Hanif F Muzaffar K Perveen K Malhi SM Simjee Sh U. Glioblastoma multiforme: a review of its epidemiology and pathogenesis through clinical presentation and treatment. Asian Pac J Cancer Prev (2017) 18(1):3–9. doi: 10.22034/APJCP.2017.18.1.318 28239999 PMC5563115

[19] Filley A Henriquez M Bhowmik T Tewari BN Rao X Wan J. Immunologic and gene expression profiles of spontaneous canine oligodendrogliomas. J Neurooncol (2018) 137(3):469–79. doi: 10.1007/s11060-018-2753-4.19 PMC5924594 29330750

[20] Jones S Beyers M Shukla M Xia F Brettin T Stevens R. TULIP: an RNA-seq-based primary tumor type prediction tool using convolutional neural networks. Cancer Inform (2022) 21:11769351221139491. doi: 10.1177/1176935122113949120 36507076 PMC9729992

[21] Megquier K Genereux DP Hekman J Swofford R Turner-Maier J Johnson J. BarkBase: epigenomic annotation of canine genomes. Genes (Basel) (2019) 10(6):433. doi: 10.3390/genes1006043321 31181663 PMC6627511

[22] Colaprico A Silva TC Olsen C Garofano L Cava C Garolini D. TCGAbiolinks: an R/Bioconductor package for integrative analysis of TCGA data. Nucleic Acids Res (2016) 44(8):e71. doi: 10.1093/nar/gkv150722 26704973 PMC4856967

[23] Mounir M Lucchetta M Silva TC Olsen C Bontempi G Chen X. New functionalities in the TCGAbiolinks package for the study and integration of cancer data from GDC and GTEx. PloS Comput Biol (2019) 15(3):e1006701. doi: 10.1371/journal.pcbi.100670123 30835723 PMC6420023

[24] Zhang Z Hernandez K Savage J Li S Miller D Agrawal S. Uniform genomic data analysis in the NCI genomic data commons. Nat Commun (2021) 12(1):1226. doi: 10.1038/s41467-021-21254-924 33619257 PMC7900240

[25] Dobin A Davis CA Schlesinger F Drenkow J Zaleski C Jha S. STAR: ultrafast universal RNA-seq aligner. Bioinformatics (2013) 29(1):15–21. doi: 10.1093/bioinformatics/bts63525 23104886 PMC3530905

[26] Anders S Pyl PT Huber W. HTSeq–a Python framework to work with high-throughput sequencing data. Bioinformatics (2015) 31(2):166–9. doi: 10.1093/bioinformatics/btu638 PMC4287950 25260700

[27] Durinck S Moreau Y Kasprzyk A Davis S De Moor B Brazma A. BioMart and bioconductor: a powerful link between biological databases and microarray data analysis. Bioinformatics (2005) 21(16):3439–40. doi: 10.1093/bioinformatics/bti525 16082012

[28] Durinck S Spellman PT Birney E Huber W. Mapping identifiers for the integration of genomic datasets with the R/Bioconductor package biomaRt. Nat Protoc (2009) 4(8):1184–91. doi: 10.1038/nprot.2009.97 PMC3159387 19617889

[29] Krijthe JH. Rtsne: T-distributed stochastic neighbor embedding using Barnes-hut implementation. In: R package version 0.13 (2015). Available at: https://github.com/jkrijthe/Rtsne.

[30] Love MI Huber W Anders S. Moderated estimation of fold change and dispersion for RNA-seq data with DESeq2. Genome Biol (2014) 15(12):550. doi: 10.1186/s13059-014-0550-8 25516281 PMC4302049

[31] Robinson MD McCarthy DJ Smyth GK. edgeR: a bioconductor package for differential expression analysis of digital gene expression data. Bioinformatics (2010) 26(1):139–40. doi: 10.1093/bioinformatics/btp616 PMC2796818 19910308

[32] McCarthy DJ Chen Y Smyth GK. Differential expression analysis of multifactor RNA-seq experiments with respect to biological variation. Nucleic Acids Res (2012) 40(10):4288–97. doi: 10.1093/nar/gks042 PMC3378882 22287627

[33] Chen Y Lun AT Smyth GK. From reads to genes to pathways: differential expression analysis of RNA-seq experiments using rsubread and the edgeR quasi-likelihood pipeline. F1000Res (2016) 5:1438. doi: 10.12688/f1000research.8987.2 27508061 PMC4934518

[34] Wickham H. ggplot2: elegant graphics for data analysis. New York: Springer-Verlag (2016).

[35] Amberger JS Bocchini CA Schiettecatte F Scott AF Hamosh A. OMIM. org: online mendelian inheritance in man (OMIM®), an online catalog of human genes and genetic disorders. Nucleic Acids Res (2015) 43(D1):D789–98. doi: 10.1093/nar/gku1205 PMC4383985 25428349

[36] Amberger JS Bocchini CA Scott AF Hamosh A. OMIM.org: leveraging knowledge across phenotype-gene relationships. Nucleic Acids Res (2019) 47(D1):D1038–D43. doi: 10.1093/nar/gky1151 PMC6323937 30445645

[37] Gu Z Eils R Schlesner M. Complex heatmaps reveal patterns and correlations in multidimensional genomic data. Bioinformatics (2016) 32(18):2847–9. doi: 10.1093/bioinformatics/btw313 27207943

[38] Mootha VK Lindgren CM Eriksson KF Subramanian A Sihag S Lehar J. PGC-1alpha-responsive genes involved in oxidative phosphorylation are coordinately downregulated in human diabetes. Nat Genet (2003) 34(3):267–73. doi: 10.1038/ng1180 12808457

[39] Subramanian A Tamayo P Mootha VK Mukherjee S Ebert BL Gillette MA. Gene set enrichment analysis: a knowledge-based approach for interpreting genome-wide expression profiles. Proc Natl Acad Sci U.S.A. (2005) 102(43):15545–50. doi: 10.1073/pnas.0506580102 PMC1239896 16199517

[40] Dhawan D Ramos-Vara JA Utturkar SM Ruple A Tersey SA Nelson JB. Identification of a naturally-occurring canine model for early detection and intervention research in high grade urothelial carcinoma. Front Oncol (2022) 12:101196. doi: 10.3389/fonc.2022.101196 PMC9692095 36439482

[41] Dhawan D Hahn NM Ramos-Vara JA Knapp DW. Naturally-occurring canine invasive urothelial carcinoma harbors luminal and basal transcriptional subtypes found in human muscle invasive bladder cancer. PloS Genet (2018) 14(8):e1007571. doi: 10.1371/journal.pgen.1007571 30089113 PMC6101404

[42] Cancer Genome Atlas Research N Brat DJ Verhaak RG Aldape KD Yung WK Salama SR. Comprehensive, integrative genomic analysis of diffuse lower-grade gliomas. N Engl J Med (2015) 372(26):2481–98. doi: 10.1056/NEJMoa1402121 PMC4530011 26061751

