# Supplementary material for: cTULIP: application of a human-based RNA-seq primary tumor classification tool for cross-species primary tumor classification in canine
Source: Front Oncol. 2023 Jul 20;13:1216892. doi: 10.3389/fonc.2023.1216892 (PMC10397722; doi:10.3389/fonc.2023.1216892)
Supplement: Supplementary file 1 [file DataSheet_1.pdf]

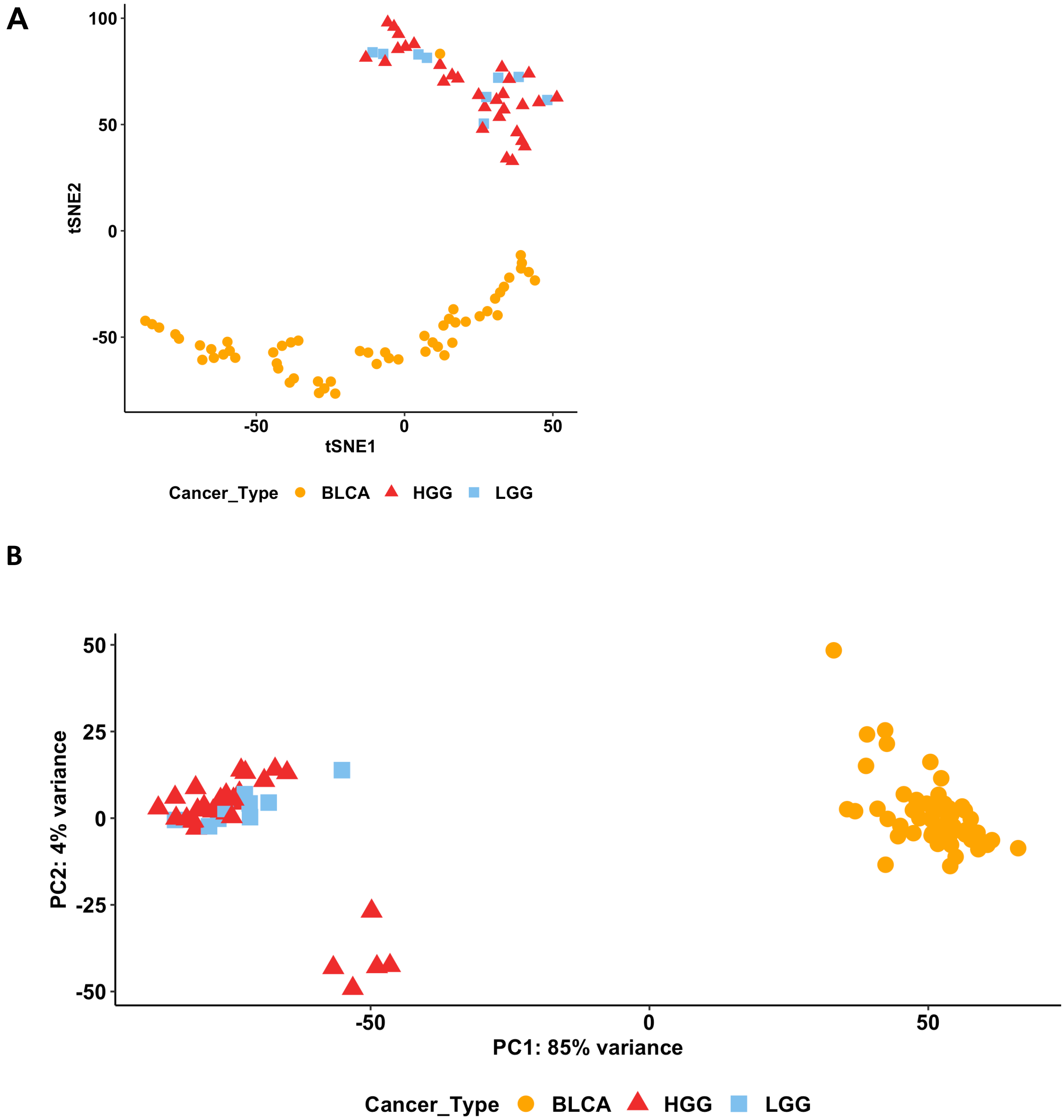

Supplementary Figure 1: Feature selection for modeling.

(A) t-SNE analysis of three cancer types in canine datasets. 15860 canine genes that have one-to-one human homologs in GDC were used. BLCA = Bladder cancer, LGG = Low-grade glioma, HGG = High-grade glioma.

(B) Principal component analysis of canine bladder cancer and glioma datasets. 15860 canine genes that have one-to-one human homologs in GDC were included. Top 500 genes exhibiting the highest row variance were used in this analysis . BLCA = Bladder cancer, LGG = Low-grade glioma, HGG = High-grade glioma.

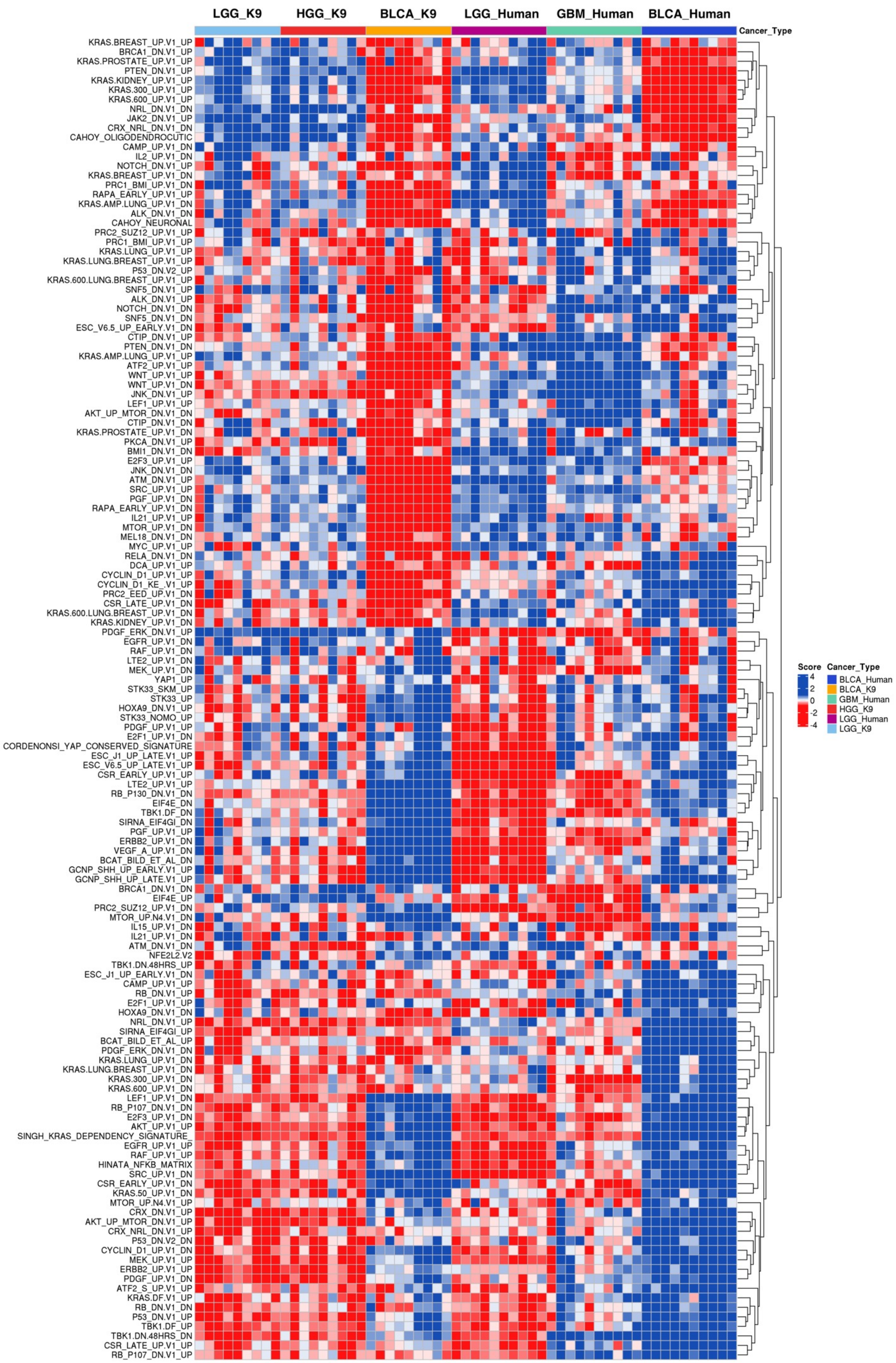
LGG_K9
HGG_K9
BLCA_K9
LGG_Human
GBM_Human
BLCA_Human
Cancer_Type
KRAS.BREAST_UP.V1_UP
BRCA1_DN.V1_UP
KRAS.PROSTATE_UP.V1_UP
PTEN_DN.V1_UP
KRAS.KIDNEY_UP.V1_UP
KRAS.300_UP.V1_UP
KRAS.600_UP.V1_UP
NRL_DN.V1_DN
JAK2_DN.V1_UP
CRX_NRL_DN.V1_DN
CAHOY_OLIGODENDROCUTIC
CAMP_UP.V1_DN
IL2_UP.V1_DN
NOTCH_DN.V1_UP
KRAS.BREAST_UP.V1_DN
PRC1_BMI_UP.V1_DN
RAPA_EARLY_UP.V1_UP
KRAS.AMP.LUNG_UP.V1_DN
ALK_DN.V1_DN
CAHOY_NEURONAL
PRC2_SUZ12_UP.V1_UP
PRC1_BMI_UP.V1_UP
KRAS.LUNG_UP.V1_UP
KRAS.LUNG.BREAST_UP.V1_UP
P53_DN.V2_UP
KRAS.600.LUNG.BREAST_UP.V1_UP
SNF5_DN.V1_UP
ALK_DN.V1_UP
NOTCH_DN.V1_DN
SNF5_DN.V1_DN
ESC_V6.5_UP_EARLY.V1_DN
CTIP_DN.V1_UP
PTEN_DN.V1_DN
KRAS.AMP.LUNG_UP.V1_UP
ATF2_UP.V1_UP
WNT_UP.V1_UP
WNT_UP.V1_DN
JNK_DN.V1_UP
LEF1_UP.V1_UP
AKT_UP_MTOR_DN.V1_DN
CTIP_DN.V1_DN
KRAS.PROSTATE_UP.V1_DN
PKCA_DN.V1_UP
BMI1_DN.V1_DN
E2F3_UP.V1_UP
JNK_DN.V1_DN
ATM_DN.V1_UP
SRC_UP.V1_UP
PGF_UP.V1_DN
RAPA_EARLY_UP.V1_DN
IL21_UP.V1_UP
MTOR_UP.V1_DN
MEL18_DN.V1_DN
MYC_UP.V1_UP
RELA_DN.V1_DN
DCA_UP.V1_UP
CYCLIN_D1_UP.V1_UP
CYCLIN_D1_KE_.V1_UP
PRC2_EED_UP.V1_DN
CSR_LATE_UP.V1_DN
KRAS.600.LUNG.BREAST_UP.V1_DN
KRAS.KIDNEY_UP.V1_DN
PDGF_ERK_DN.V1_UP
EGFR_UP.V1_DN
RAF_UP.V1_DN
LTE2_UP.V1_DN
MEK_UP.V1_DN
YAP1_UP
STK33_SKM_UP
STK33_UP
HOXA9_DN.V1_UP
STK33_NOMO_UP
PDGF_UP.V1_UP
E2F1_UP.V1_DN
CORDENONSI_YAP_CONSERVED_SIGNATURE
ESC_J1_UP_LATE.V1_UP
ESC_V6.5_UP_LATE.V1_UP
CSR_EARLY_UP.V1_UP
LTE2_UP.V1_UP
RB_P130_DN.V1_DN
EIF4E_DN
TBK1.DF_DN
SIRNA_EIF4GI_DN
PGF_UP.V1_UP
ERBB2_UP.V1_DN
VEGF_A_UP.V1_DN
BCAT_BILD_ET_AL_DN
GCNP_SHH_UP_EARLY.V1_UP
GCNP_SHH_UP_LATE.V1_UP
BRCA1_DN.V1_DN
EIF4E_UP
PRC2_SUZ12_UP.V1_DN
MTOR_UP.N4.V1_DN
IL15_UP.V1_DN
IL21_UP.V1_DN
ATM_DN.V1_DN
NFE2L2.V2
TBK1.DN.48HRS_UP
ESC_J1_UP_EARLY.V1_DN
CAMP_UP.V1_UP
RB_DN.V1_UP
E2F1_UP.V1_UP
HOXA9_DN.V1_DN
NRL_DN.V1_UP
SIRNA_EIF4GI_UP
BCAT_BILD_ET_AL_UP
PDGF_ERK_DN.V1_DN
KRAS.LUNG_UP.V1_DN
KRAS.LUNG.BREAST_UP.V1_DN
KRAS.300_UP.V1_DN
KRAS.600_UP.V1_DN
LEF1_UP.V1_DN
RB_P107_DN.V1_DN
E2F3_UP.V1_DN
AKT_UP.V1_UP
SINGH_KRAS_DEPENDENCY_SIGNATURE_
EGFR_UP.V1_UP
RAF_UP.V1_UP
HINATA_NFKB_MATRIX
SRC_UP.V1_DN
CSR_EARLY_UP.V1_DN
KRAS.50_UP.V1_DN
MTOR_UP.N4.V1_UP
CRX_DN.V1_UP
AKT_UP_MTOR_DN.V1_UP
CRX_NRL_DN.V1_UP
P53_DN.V2_DN
CYCLIN_D1_UP.V1_DN
MEK_UP.V1_UP
ERBB2_UP.V1_UP
PDGF_UP.V1_DN
ATF2_S_UP.V1_UP
KRAS.DF.V1_UP
RB_DN.V1_DN
P53_DN.V1_UP
TBK1.DF_UP
TBK1.DN.48HRS_DN
CSR_LATE_UP.V1_UP
RB_P107_DN.V1_UP
Score
4
2
0
-2
-4
Cancer_Type
BLCA_Human
BLCA_K9
GBM_Human
HGG_K9
LGG_Human
LGG_K9

Supplementary Figure 2: Single-sample Gene Set Enrichment Analysis of canine RNA-seq data. TPMs were used to perform ssGSEA analysis. Annotated oncogenic signature gene sets (c6) in MSigDB database were included in the initial analysis. The heat map of derived ssGSEA scores of 9 randomly selected samples of each canine cancer type (K9) and 10 randomly selected randomly sample (Human) from each human primary tumor type was plotted. Enriched gene sets in either glioma or bladder cancer were determined by having at least 2/3 of total sample size with an FDR threshold of < 0.05. The union of enriched gene sets in each cancer type results in 160 gene sets as indicated. BLCA = Bladder cancer, LGG = Low-grade glioma, HGG = High-grade glioma.

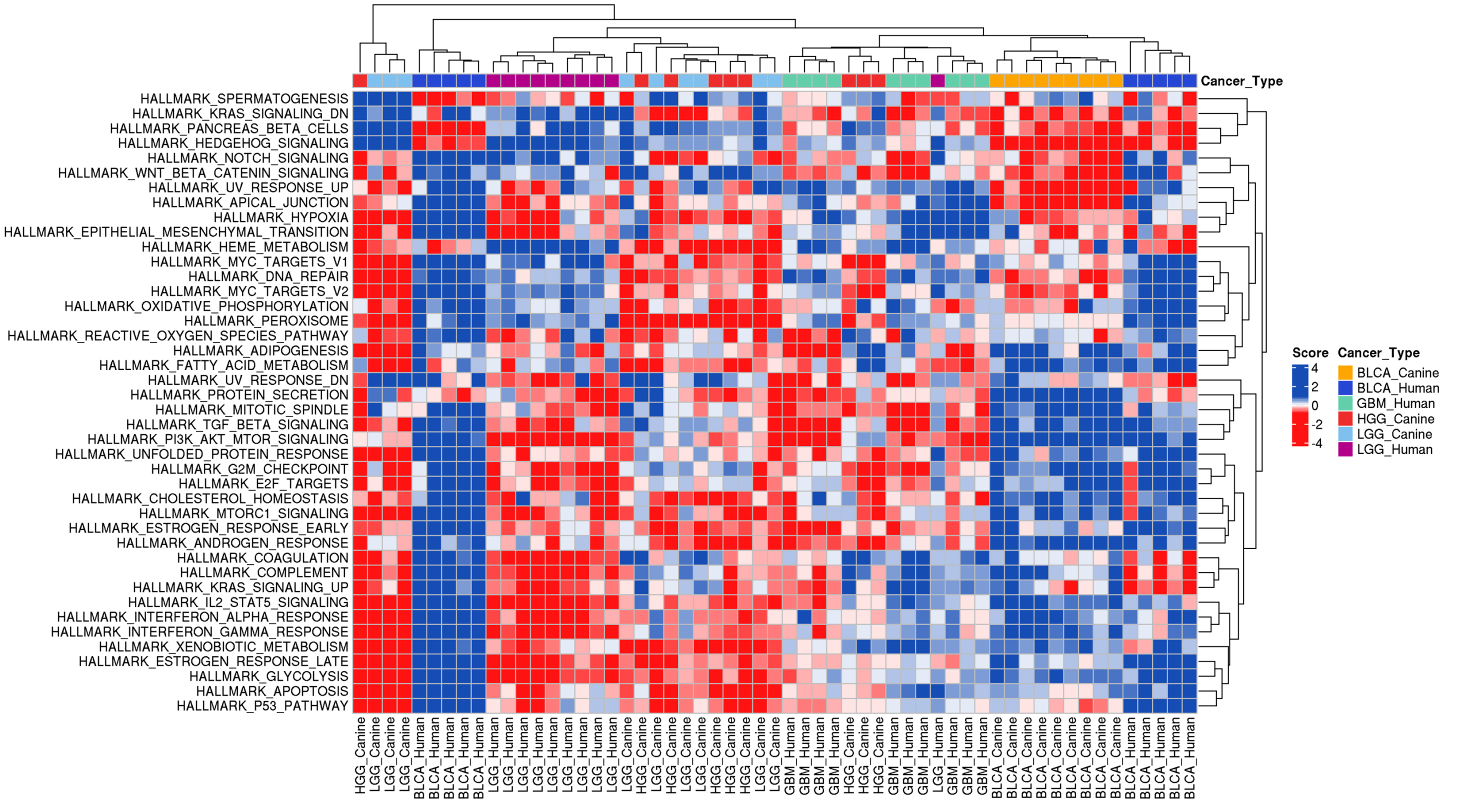

Supplementary Figure 3: Single-sample Gene Set Enrichment Analysis of canine RNA-seq data and human RNA-seq data. TPMs were used to perform ssGSEA analysis. Annotated hallmark gene sets (h) in MSigDB database were included in the initial analysis. The heat map of derived ssGSEA scores of 9 randomly selected samples of each canine cancer type (Canine) and 10 randomly selected randomly sample (Human) from each human primary tumor type was plotted. Enriched gene sets in either canine glioma or canine bladder cancer were determined by having at least 2/3 of total sample size with an FDR threshold of < 0.05. The union of enriched gene sets in both cancer types results in 42 gene sets as indicated. BLCA = Bladder cancer, LGG = Low-grade glioma, GBM = Glioblastoma, HGG = High-grade glioma. The heat map was clustered based on both columns and rows.
